# Lamotrigine-Induced Toxic Epidermal Necrolysis (Lyell’s Syndrome): A Case Report and Literature Review

**DOI:** 10.7759/cureus.84929

**Published:** 2025-05-27

**Authors:** Mourad Nafaa, Filda Messal, Amal Miqdadi, Mostapha Noussair, Belyamani Lahcen

**Affiliations:** 1 Emergency Department, Faculty of Medicine, Mohammed VI University of Health Sciences, Cheikh Khalifa International University Hospital, Casablanca, MAR; 2 Family Medicine, Hospital de La Princesa, Madrid, ESP; 3 Nuclear Medicine, Mohammed VI University of Health Sciences, Cheikh Khalifa International University, Casablanca, MAR; 4 Emergency Department, Mohamed V Military Hospital, Rabat, MAR

**Keywords:** allergic skin reactions, emergency, intensive care unit stay, lamotrigine, lyell syndrome, toxic epidermal necrolysis

## Abstract

Lyell's syndrome, also known as toxic epidermal necrolysis (TEN), is a rare and severe adverse drug reaction characterized by widespread skin detachment, mucosal involvement, and high mortality. We present the case of a 24-year-old female who developed TEN after the recent initiation of lamotrigine for bipolar disorder. The patient exhibited rapidly progressing skin blistering and detachment affecting over 30% of the body surface area, along with mucosal lesions. Lamotrigine was immediately discontinued, and the patient received intensive supportive treatment in a specialized care unit, resulting in a favorable recovery.

This case emphasizes the importance of early recognition of TEN and immediate withdrawal of the offending agent. It also highlights the need for heightened clinical awareness when prescribing lamotrigine, especially during the initial titration phase. We aim to contribute to the growing literature on lamotrigine-induced TEN and underline the value of timely diagnosis and multidisciplinary management in improving patient outcomes.

## Introduction

Lyell's syndrome is an unpredictable, severe, and potentially fatal drug allergy. However, it is rare, with an incidence of 1 to 2 cases per million people per year [1]. The literature reports a slight female predominance, with a sex ratio of 0.6 [2]. In Morocco, since the establishment of the Poison Control and Pharmacovigilance Center in 1989 until July 2016, 122 cases of Lyell's syndrome have been reported, with a strong female predominance. This national data underscores the rarity of the condition in our context and highlights the importance of documenting individual cases like ours to raise awareness and contribute to regional pharmacovigilance efforts [3].

No age group is spared, but the incidence is higher in the elderly, likely due to increased medication consumption in this population. Additionally, individuals with AIDS, systemic lupus erythematosus, or those who have undergone allogeneic bone marrow transplantation are at higher risk [2].

Histologically, toxic epidermal necrolysis (TEN) is characterized by necrosis of keratinocytes and disruption of the dermoepidermal junction [4]. Several genetic, immunological, and viral factors are presumed to be involved in the pathophysiology of Lyell's syndrome, though they remain poorly understood [5].

We report a case of TEN following the oral intake of lamotrigine, an antiepileptic drug, which occurred at usual therapeutic doses.

## Case presentation

Our patient was a 24-year-old woman with stable bipolar disorder under treatment, with no other medical history. She was admitted to the ED for the sudden onset of skin lesions covering her entire body, sparing the lower limbs. On questioning, these lesions coincided with a recent change in her psychiatric treatment one week prior: the patient had started taking Lamictal, Fluoxetine, and Largactil. The symptoms initially began with a limited rash on the lower limbs after taking the medication, which was not discontinued following the appearance of the rash.

**Figure 1:**
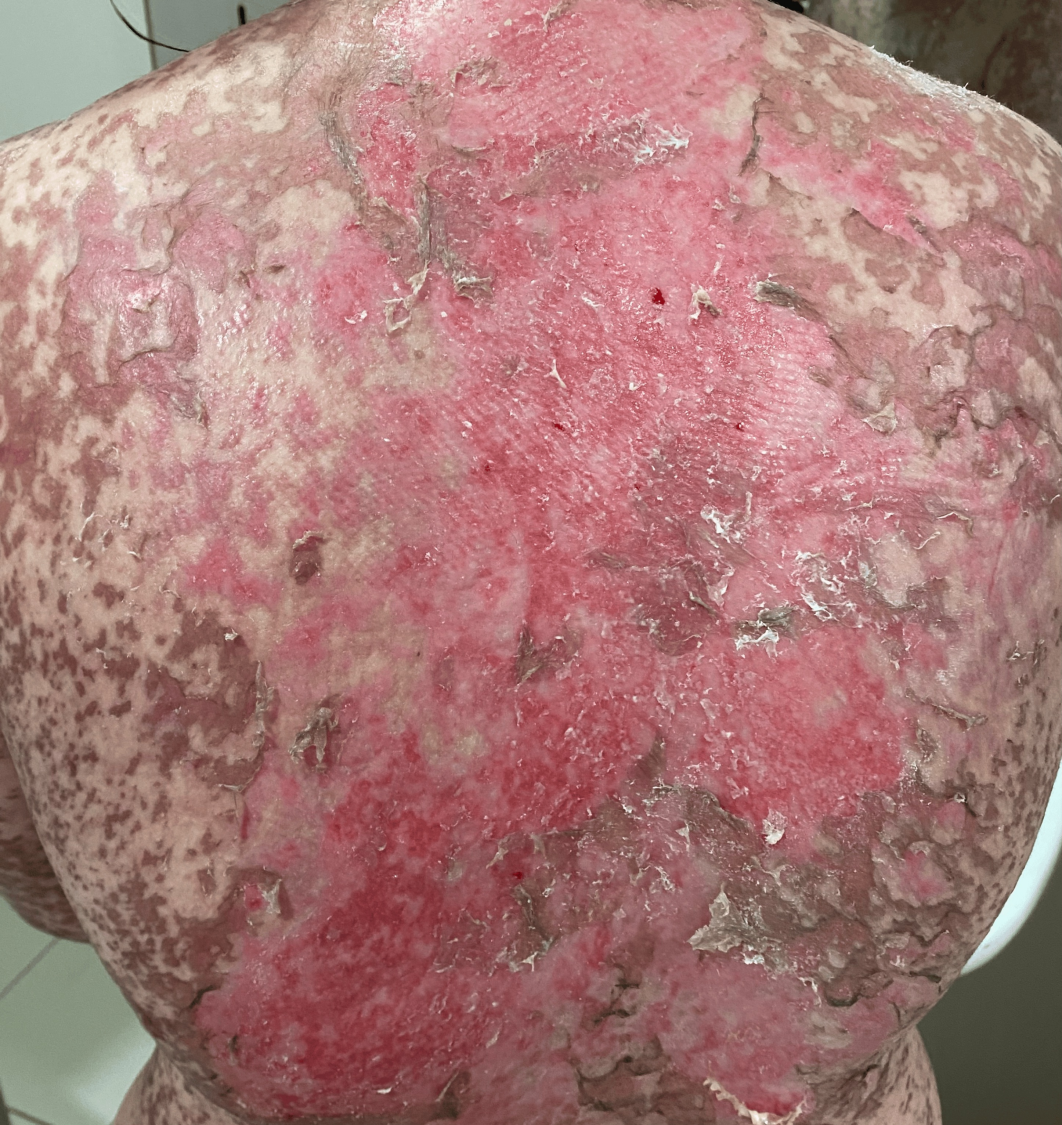
Torso showing lesions with positive Nikolsky’s sign.

**Figure 2:**
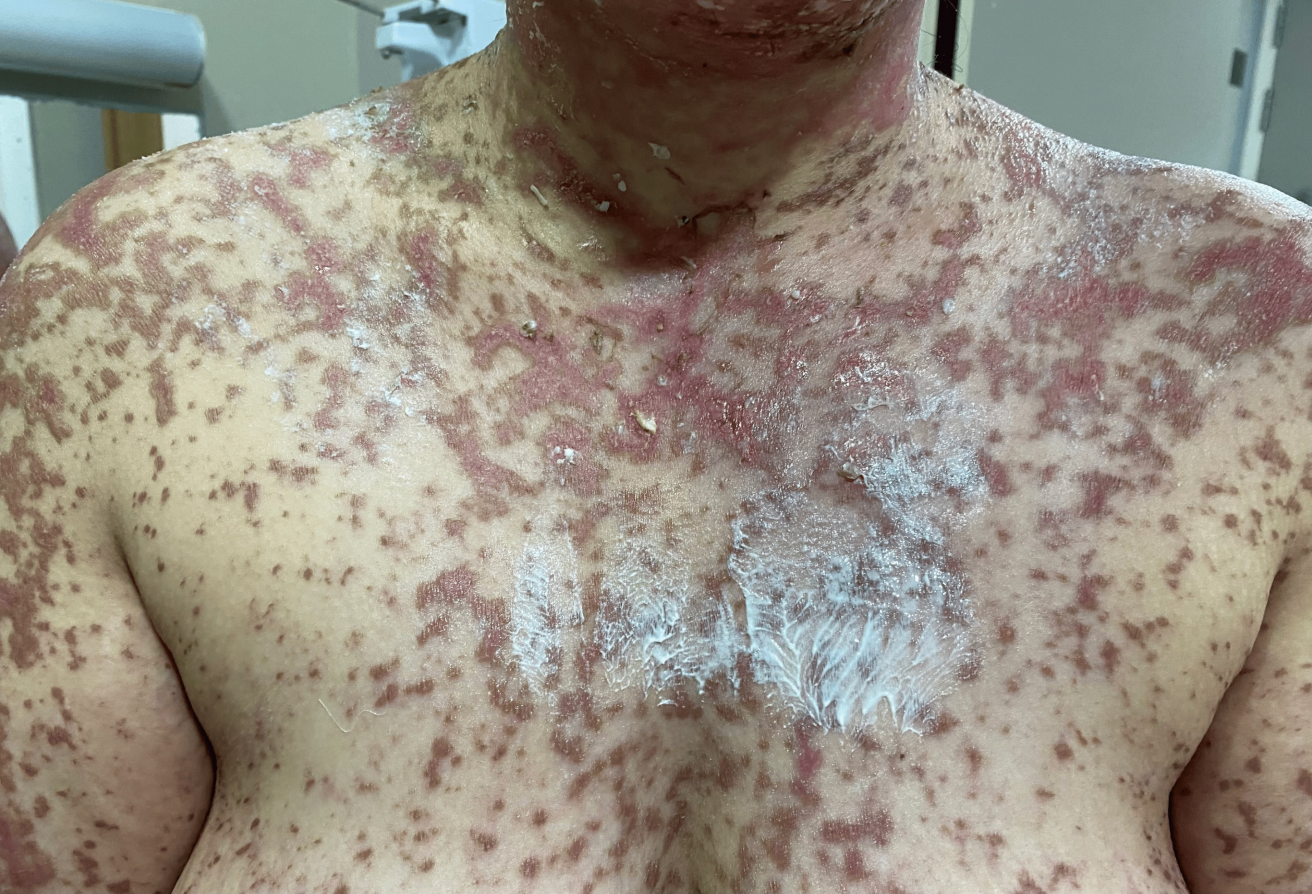
Thorax with erythematous and livid macules.

**Figure 3:**
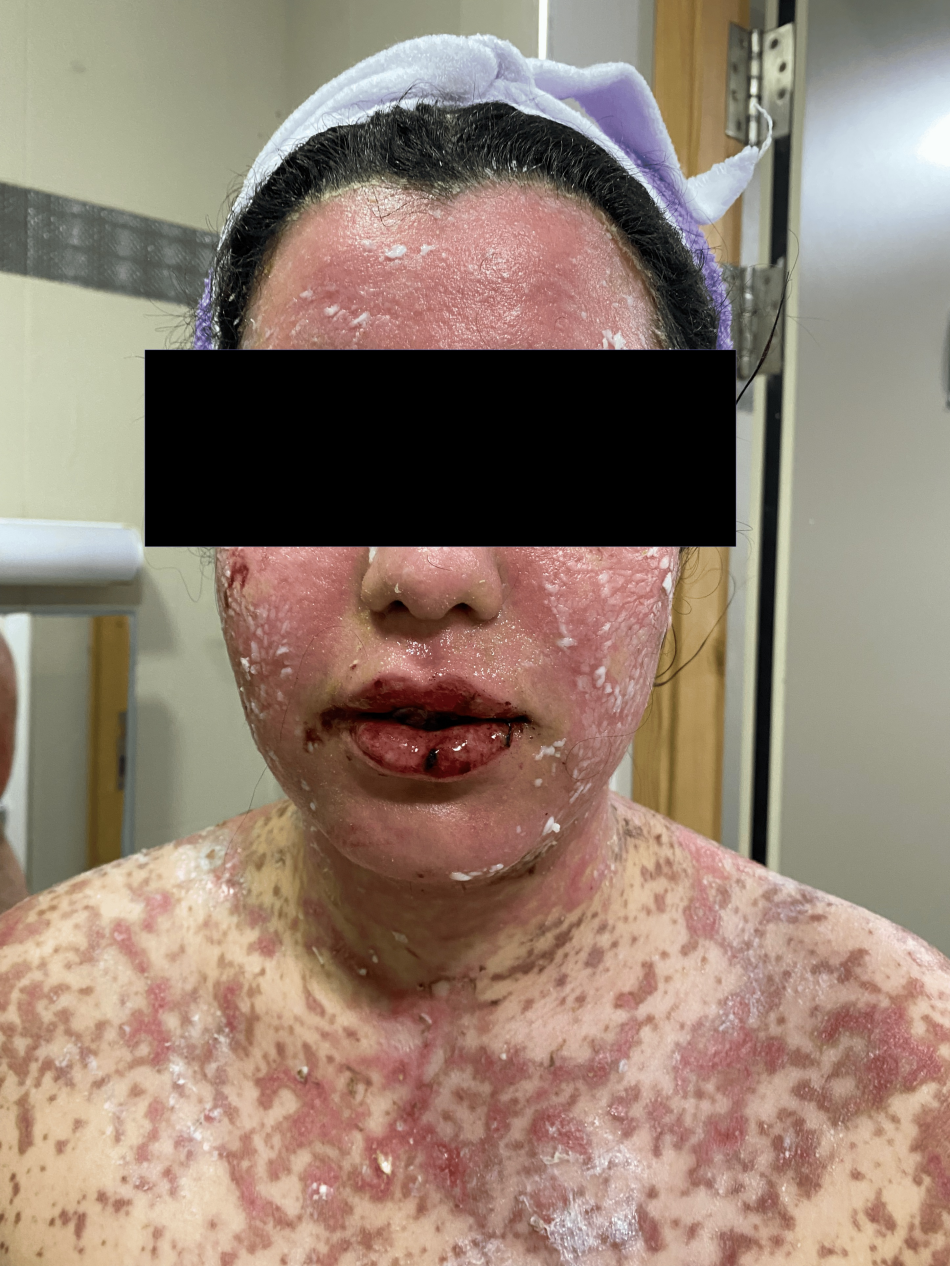
Facial lesions with oral mucosal erosion.

**Figure 4:**
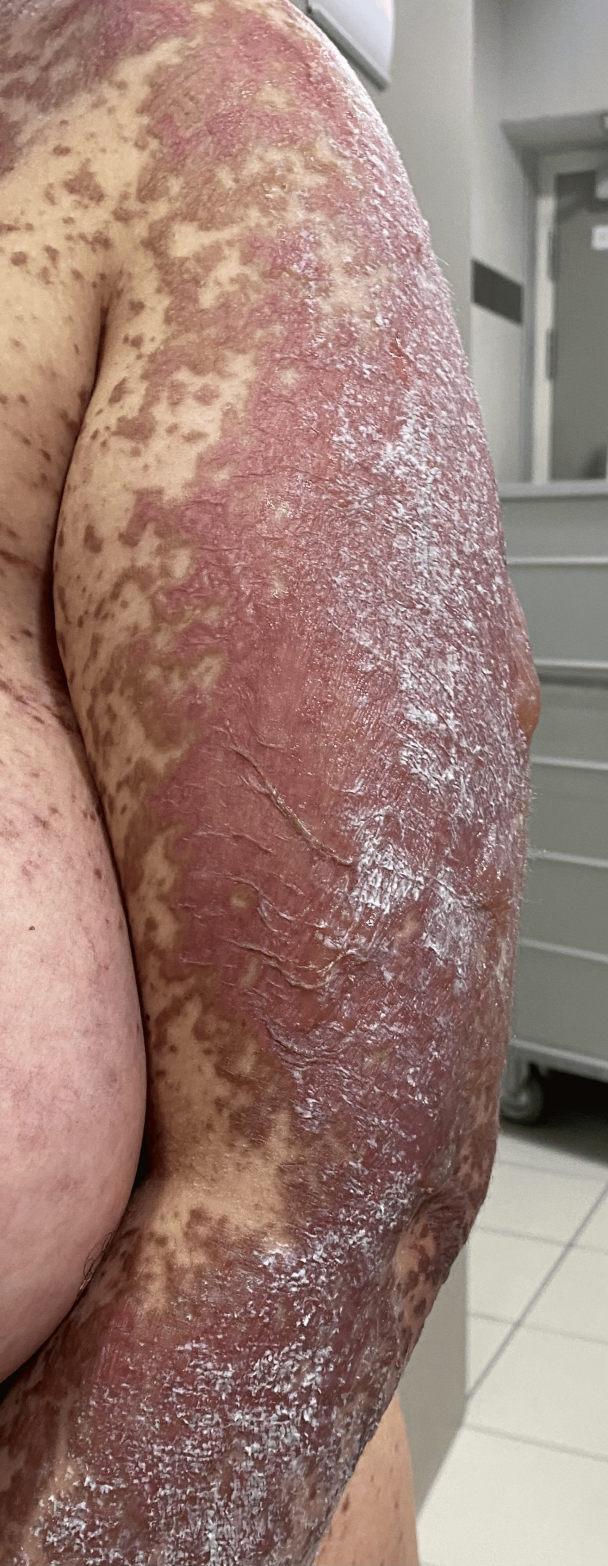
Left arm with typical lesions of toxic epidermal necrolysis (TEN).

**Figure 5:**
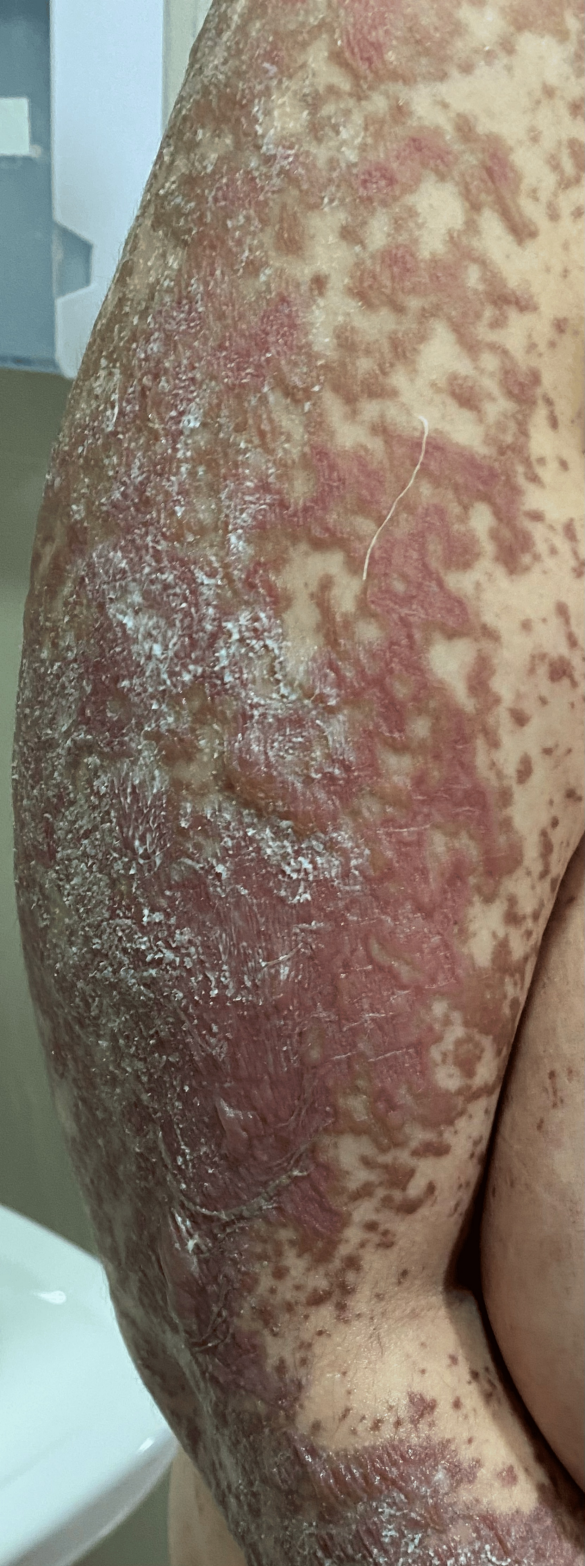
Right arm with typical lesions of toxic epidermal necrolysis (TEN).

On admission, the patient was conscious, with a blood pressure of 120/70 mm Hg, heart rate of 86 beats per minute, temperature of 37°C, and oxygen saturation of 95% in ambient air. Clinical examination revealed a rash on the trunk, upper limbs, and face (Figures 1-5). The skin examination showed erythematous macular and maculopapular lesions of varying sizes on the face, back, and upper limbs, with a positive Nikolsky sign (Figure 1). This sign, characterized by the detachment of the superficial epidermis from the dermis with gentle lateral pressure on apparently unaffected skin, reflects the severe epidermal necrosis and loss of cell-to-cell adhesion seen in TEN and Stevens-Johnson Syndrome (SJS), a related but less extensive condition within the same spectrum of severe mucocutaneous reactions, typically involving less than 10% of total body surface area (TBSA) detachment [6]. In our case, TBSA involvement was evaluated at 32.5%, consistent with a diagnosis of TEN. Examination of the mucous membranes revealed erosions of the oral mucosa accompanied by gingivorrhagia and dysphagia secondary to pharyngitis, as well as genital and ocular ulcerations (Figure 3).

**Figure 6:**
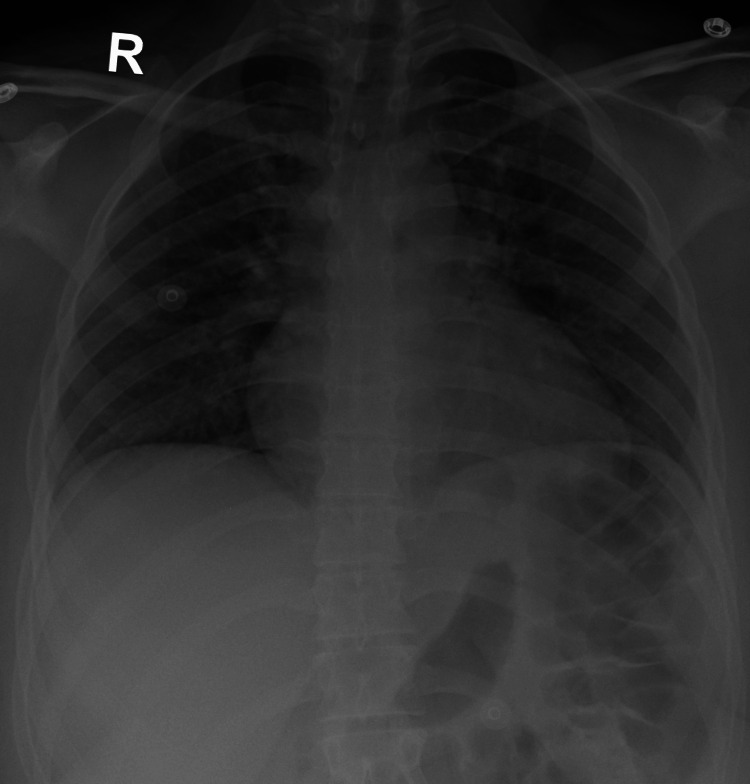
Normal chest X-rays.

Radiographic assessments did not show any pulmonary involvement (Figure 6). Laboratory results showed leukocytosis at 17,990 cells/mm³ (normal range 3,800-11,000), predominantly neutrophils at 15,360 cells/mm³ (normal range 1,400-7,700), all evolving in the context of an afebrile condition. Renal function remained preserved throughout her hospital stay, and electrophoresis revealed a profile consistent with a significant inflammatory syndrome (Table 1). A urine drug screen at admission was negative. Urine culture showed colonization by *Escherichia coli* without associated infectious signs and was sensitive on antibiotic susceptibility testing.

**Table 1:** Ionogram results within normal ranges. Na⁺: Sodium; K⁺: Potassium; Cl⁻: Chloride; Ca²⁺: Calcium; BUN: Blood Urea Nitrogen.

Electrolytes	Patient	Normal Range
Na⁺ (mEq/L)	137	136-145
K⁺ (mEq/L)	4	3.5-5.1
Cl⁻ (mEq/L)	105	98-107
Bicarbonate (mEq/L)	22	22-30
Protein (g/L)	79	64-83
Ca²⁺ (mg/L)	101	85-101
BUN (g/L)	0.26	0.15-0.45
Creatinine (mg/L)	8.9	6-12

The survival prognosis during an episode of TEN can be assessed using the SCORTEN, a validated severity-of-illness score specifically designed to predict mortality in patients with TEN. It includes seven independent clinical and laboratory criteria, each contributing one point if present, with the total score corresponding to an estimated mortality risk. SCORTEN is typically calculated on the first and third days of hospitalization [7]. In our case, SCORTEN was calculated on admission, yielding a score of 1, which corresponds to an estimated mortality risk of 3.2%.

The mainstay of treatment for TEN includes immediate discontinuation of the offending drug, intensive supportive care, ideally in a burn unit or intensive care setting, and consideration of immunomodulatory therapies such as intravenous immunoglobulin (IVIG), systemic corticosteroids, or cyclosporine in select cases [8].

A multidisciplinary approach is essential in the management of TEN. Intensive care specialists provide hemodynamic support and monitor for systemic complications; dermatologists guide wound care and assess disease severity; ophthalmologists manage ocular involvement to prevent long-term visual impairment; and psychiatrists address the psychological impact of the illness and any underlying psychiatric conditions, especially in cases where the triggering medication was prescribed for mental health disorders. This collaborative approach improves outcomes and reduces long-term complications [9].

In the case of our patient, following guidelines, the immediate cessation of the implicated medication and a change in her treatment for bipolar disorder were initiated. She was prescribed Seroquel 200 mg and Teralithe 250 mg, with monitoring of lithium levels.

Initial supportive management included hydration with 3 liters of 5% glucose solution, along with hydroelectrolytic supplementation and parenteral nutrition (Oliclinomel®) at a rate of 1 liter per day. The patient did not present with laboratory signs of dehydration or electrolyte imbalance; therefore, the ionogram values in Table 1 confirmed metabolic stability and did not influence specific therapeutic decisions.

Treatment for ocular lesions involved antibiotic eye drops, artificial tears, and healing agents. Skin care included the use of Biafine®, petroleum jelly, twice-daily dressing changes with warm baths, and the application of sterile emollients to detached areas, followed by covering with sterile dressings. Mouth rinses were also employed.

Thromboembolic disease prevention was carried out with 0.4 mL of sodium enoxaparin administered every 24 hours, and stress ulcer prevention was implemented with 40 mg of omeprazole every 24 hours. Analgesia was systematically provided with paracetamol at a dosage of 1 gram every 6 hours. The use of morphine was limited due to the patient’s psychiatric history. Corticosteroid therapy was initiated upon admission with methylprednisolone at a dose of 120 mg/day.

On the second day of hospitalization, an increase in CRP to 135.9 mg/L (normal range <8) and LDH to 255 IU/L (normal range 80-230) was observed. Antibiotic therapy was initiated with Augmentin 1 g/day.

On the fourth day of hospitalization, a central venous catheter was placed to initiate treatment with intravenous immunoglobulins (Tegeline®) at a dosage of 7 vials of 5 g/100 mL per day for five consecutive days. The initiation of this therapy was delayed due to the patient’s financial constraints.

**Figure 7:**
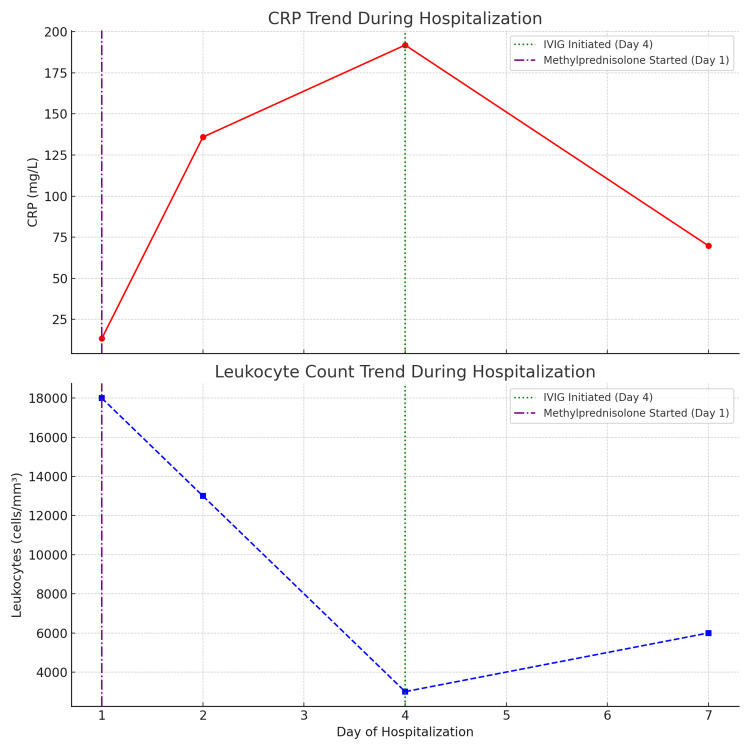
Trends in CRP and leukocyte count during hospitalization. This figure illustrates the temporal progression of CRP and leukocyte levels in the patient over the course of hospitalization. A significant rise in CRP was observed, peaking on day 4 at 191.9 mg/L, followed by a notable decrease to 69.7 mg/L by discharge (day 7). Leukocyte counts showed a similar trend, decreasing from 18,000 cells/mm³ on admission to 3,000 cells/mm³ by day 4, and gradually recovering to 6,000 cells/mm³ by day 7. Intravenous immunoglobulin (IVIG) therapy was initiated on day 4, coinciding with the peak of inflammation, while methylprednisolone was started on day 1. These interventions appear to correlate with the subsequent clinical and biological improvements observed in the patient.

A gradual decline in inflammatory markers was noted starting from the third day of IVIG administration. The patient’s overall clinical improvement was accompanied by a progressive normalization of laboratory values. Both CRP and leukocyte counts showed a significant decrease over the course of hospitalization, correlating with the introduction of immunoglobulin therapy (Figure 7). The progression was also marked by accelerated lesion healing following the initiation of treatment.

## Discussion

TEN, also known as Lyell's syndrome, is a rare but severe medical condition. It is characterized by the formation of blisters on the skin and mucous membranes, resulting from blistering beneath the epidermis and necrosis of keratinocytes [4]. These manifestations typically occur 1 to 3 weeks after the initiation of the implicated medication [10]. 

Mortality in TEN remains high despite optimal infection control and wound care. Diagnosis is primarily clinical, with a fatal outcome in approximately 22% of cases. Among survivors, 80% experience sequelae, mainly ocular, genital, cutaneous, and bronchial complications [5]. The SCORTEN scale is a severity-of-illness scoring system, in which increasing values indicate an elevated risk of mortality (Table 2) [7,11].

**Table 2:** Severity-of-illness score for toxic epidermal necrolysis (SCORTEN). More risk factors indicate a higher score and a higher mortality rate (%) as follows: 0-1 = 3.2% (CI: 0.1 to 16.7%); 2 = 12.1% (CI: 5.4 to 22.5%); 3 = 35.3% (CI: 19.8 to 53.5%); 4 = 58.3% (CI: 36.6 to 77.9%); ≥ 5 = >90% (CI: 55.5 to 99.8%).

Risk Factor	Score 0	Score 1
Age	< 40 years	≥ 40 years
Associated Cancer	No	Yes
Heart Rate (beats/minute)	< 120	≥ 120
Serum Blood Urea Nitrogen	≤ 28 mg/dL (≤10 mmol/L)	> 28 mg/dL (>10 mmol/L)
Detached or Compromised Body Surface Area	< 10%	≥ 10%
Serum Bicarbonate	≥ 20 mEq/L (≥20 mmol/L)	< 20 mEq/L (<20 mmol/L)
Serum Glucose	≤ 250 mg/dL (≤13.88 mmol/L)	> 250 mg/dL (>13.88 mmol/L)

The drugs most frequently implicated in Morocco, in descending order, are: anticonvulsants (phenobarbital, carbamazepine), analgesics (acetylsalicylic acid, paracetamol), NSAIDs (celecoxib, ibuprofen), the sulfamethoxazole-trimethoprim combination, allopurinol, and amoxicillin-clavulanic acid [3]. Our case of TEN followed oral intake of lamotrigine, an antiepileptic drug that, according to the literature, can cause skin eruptions in 10% of cases [2].

The immunological mechanisms underlying TEN are only partially understood, and areas of uncertainty remain. However, it is now well-established that certain Human Leukocyte Antigen (HLA) haplotypes are specifically associated with the risk of developing T-cell-mediated drug hypersensitivity to particular medications [2].

However, there are no unequivocal treatment guidelines for TEN. The use of intravenous immunoglobulins (IVIG) remains a topic of debate in the medical literature. Although the presumed effectiveness of IVIG has not yet been conclusively proven, considerable hope continues to be placed on this therapy. A consensus is needed to establish a clear and practical protocol with well-defined dosage recommendations.

Recent evidence supports the involvement of the Fas death receptor (CD95) and its ligand FasL in the pathogenesis of keratinocyte apoptosis in TEN [12,13]. The Fas receptor is a transmembrane protein belonging to the tumor necrosis factor (TNF) receptor superfamily and plays a key role in regulating programmed cell death (apoptosis). Its interaction with FasL, a type II transmembrane protein expressed on activated T cells and other immune cells, triggers a signaling cascade that induces apoptosis in Fas-bearing cells, including keratinocytes. Fas-mediated keratinocyte apoptosis, which leads to epidermal detachment in TEN, has been shown to be inhibited in vitro by monoclonal antibodies that block Fas, as well as by IVIG, which are known to contain natural anti-Fas antibodies [12].

In the literature, meta-analyses regarding the use of immunoglobulins in TEN have already been conducted. The most recent, published in December 2022 and based on a retrospective study spanning 10 years, concluded that a combination of plasmapheresis and intravenous immunoglobulins to control the disease reduced the risk of mortality fivefold, thereby improving the prognosis of TEN patients [13]. However, the high cost and ongoing uncertainty regarding the effectiveness of intravenous immunoglobulin make its use still controversial. It is important to note that some failures in this therapeutic protocol may be partly due to insufficient dosage or delayed initiation of treatment [12].

On the other hand, the use of corticosteroids remains debated in comparison to supportive treatment alone [14,15]. The administration of systemic corticosteroids such as methylprednisolone has been associated with reduced inflammation and improved outcomes in some cases of TEN, although their use remains controversial and should be carefully evaluated based on clinical judgment [16]. In our case, the use of methylprednisolone appears to have played a favorable role in the patient's recovery (Figure 7).

## Conclusions

This case underscores the critical importance of early recognition and a multidisciplinary approach in managing TEN, involving intensive care for supportive measures, strict aseptic technique, fluid and nutritional management, local wound care, psychiatric support, and targeted immunosuppressive therapy.

In particular, our patient’s favorable response to immunoglobulin treatment suggests that this therapy may accelerate skin healing and improve outcomes in TEN. Given the ongoing debate about the efficacy and risks of immunoglobulins, this report adds valuable clinical evidence supporting their use. Further studies are needed to better define the role of immunomodulatory treatments in reducing morbidity and mortality in this severe drug reaction.
